# Role of Postoperative Complications in Overall Survival after Radical Resection for Gastric Cancer: A Retrospective Single-Center Analysis of 1107 Patients

**DOI:** 10.3390/cancers11121890

**Published:** 2019-11-27

**Authors:** Christian Galata, Susanne Blank, Christel Weiss, Ulrich Ronellenfitsch, Christoph Reissfelder, Julia Hardt

**Affiliations:** 1 Department of Surgery, Universitätsmedizin Mannheim, Medical Faculty Mannheim, Heidelberg University, 68167 Mannheim, Germany; christian.galata@umm.de (C.G.); susanne.blank@umm.de (S.B.); christoph.reissfelder@umm.de (C.R.); 2 Department of Medical Statistics and Biomathematics, Medical Faculty Mannheim, Heidelberg University, 68167 Mannheim, Germany; christel.weiss@medma.uni-heidelberg.de; 3 Department of Visceral, Vascular and Endocrine Surgery, University Hospital Halle (Saale), Martin-Luther-University Halle-Wittenberg, 06120 Halle (Saale), Germany; ulrich.ronellenfitsch@uk-halle.de

**Keywords:** gastric cancer, gastrectomy, complications, outcome, survival

## Abstract

*Background:* The aim of this study was to investigate the impact of postoperative complications on overall survival (OS) after radical resection for gastric cancer. *Methods:* A retrospective analysis of our institutional database for surgical patients with gastroesophageal malignancies was performed. All consecutive patients who underwent R0 resection for M0 gastric cancer between October 1972 and February 2014 were included. The impact of postoperative complications on OS was evaluated in the entire cohort and in a subgroup after exclusion of 30 day and in-hospital mortality. *Results:* A total of 1107 patients were included. In the entire cohort, both overall complications (*p* < 0.001) and major surgical complications (*p* = 0.003) were significant risk factors for decreased OS in univariable analysis. In multivariable analysis, overall complications were an independent risk factor for decreased OS (*p* < 0.001). After exclusion of patients with complication-related 30 day and in-hospital mortality, neither major surgical (*p* = 0.832) nor overall complications (*p* = 0.198) were significantly associated with decreased OS. *Conclusion:* In this study, postoperative complications influenced OS due to complication-related early postoperative deaths. In patients successfully rescued from early postoperative complications, neither overall complications nor major surgical complications were risk factors for decreased survival.

## 1. Introduction

Even today, surgery for gastric cancer remains challenging, and patients undergoing radical resection are reported to have high complication and failure-to-rescue rates [1,2]. Failure-to-rescue rates are reported to be even higher after surgery for gastric cancer than after esophageal resections [3]. Recently, several studies have reported adverse effects of postoperative complications on overall survival (OS) in these patients. Such studies have attracted particular interest as they suggest that postoperative complications have a negative impact on oncologic outcomes. However, to understand the importance of postoperative morbidity for oncologic outcomes, patients with complication-related early postoperative mortality must be critically considered. A recent systematic review and meta-analysis including 16 retrospective studies found that postoperative complications are correlated with poor prognosis after radical gastrectomy [4]. Thirteen of these studies reported effects of postoperative complications on OS, but only eight excluded influences from in-hospital death in the survival analysis. This is of particular interest because the pooled hazard ratio in this meta-analysis was notably lower after the exclusion of in-hospital mortality (1.40 vs. 1.79). Moreover, of the six studies reporting correlations between postoperative infectious complications and OS, only four excluded in-hospital mortality. Furthermore, the authors found a lower pooled hazard ratio (1.47 vs. 1.86) depending on whether in-hospital mortality was excluded from their analysis. They also reported different 95% confidence intervals (CI) for these two scenarios (1.22–2.83 for included in-hospital mortality vs. 0.90–2.40 for excluded in-hospital mortality).

In general, studies investigating the effect of postoperative morbidity on OS after surgery for gastric cancer either exclude or include patients with complication-related early postoperative deaths. Data on how the inclusion or exclusion of in-hospital mortality affects the role of postoperative complications in decreased OS within the same study population are scarce. The aim of this study was to investigate risk factors for decreased OS in patients undergoing radical resection for gastric cancer with special regard to the effect of postoperative complications. Therefore, we investigated two different cohorts, one including and one excluding patients with complication-related postoperative deaths.

## 2. Results

### 2.1. Patient Characteristics

Data of 1107 consecutive patients who underwent R0 resection for M0 gastric cancer at our institution between October 1972 and February 2014 were included in the analysis. Patient characteristics are shown in Table 1. There were more males than females (54.9% vs. 45.1%) and the median age was 65 years old. Most tumors were proximally located (60.9%) and predominantly classified as non-diffuse type (59.1%) according to the Laurén classification. The proportion of signet-ring cell carcinomas was 27.6%. As a rigorous standard at our institution, all oncologic resections with curative intent were performed by senior surgeons specialized in upper gastrointestinal surgery. During the study period, the standard approach for radical resection of gastric cancer at our department was open total or subtotal (4/5) gastrectomy with D2 lymphadenectomy (LAD). Total gastrectomy was performed in 47.1% of the cases and subtotal gastrectomy in 52.9% of the cases. Types of reconstruction after total gastrectomy were Roux-en-Y (n = 313, 28.3%), Longmire’s reconstruction (n = 143, 12.9%), Schloffer’s reconstruction (n = 69, 5.7%) and esophago-duodenostomy (n = 2, 0.2%). For subtotal gastrectomies, the type of reconstruction was documented in 489 cases (83.5%). Most patients received Billroth II procedures (total: n = 447, 40.3%; retrocolic: n = 234, 21.1%; antecolic: n = 213, 19.2%), whereas Billroth I procedures were performed in 42 patients (3.8%). The remaining 97 patients (16.5%) underwent subtotal gastrectomy without documentation of the reconstruction method. Multivisceral resections were performed in 45.9% of the patients, with splenectomies (34.2%) and cholecystectomies (10.9%) being the most common procedures.

Locally advanced tumor stages (pT2–4) were observed in 75.9% of the patients, and positive lymph nodes at the final pathology workup (pN1–3) were present in 52.1%. While pN stages were documented from the inception of the database, for over more than four decades, the extent of LAD and the number of harvested lymph nodes were not documented until 1998. However, when the 214 cases where the number of harvested lymph nodes was available were analyzed, the median number of harvested lymph nodes was 21 (17–27), indicating an adequate extent of LAD. Of the 136 patients where D1–3 LAD was documented, the vast majority underwent D2 LAD (n = 120, 86.9%) while D1 LAD and D3 LAD was performed in 10 (7.3%) and 8 (5.8%) patients, respectively. Data on the American Society of Anesthesiologists (ASA) physical status classification system was not documented before 2008. When patients with data on ASA grading (n = 46) were evaluated, 13.0% were categorized as ASA I, 37.0% as ASA II, 45.7% as ASA III and 4.3% as ASA IV.

### 2.2. Postoperative Outcomes

**Figure 1:**
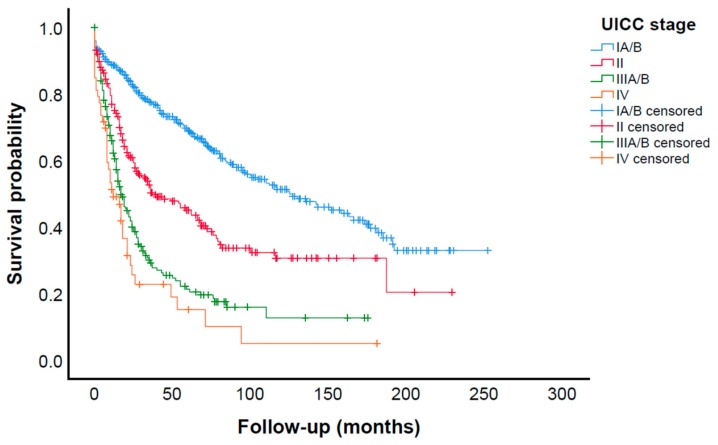
Kaplan–Meier survival curves. OS was significantly different across UICC stages (*p* < 0.001). Median OS of all patients was 61 (95% CI: 50.05–71.95) months. OS: overall survival; UICC: Union Internationale Contre le Cancer.

Postoperative outcomes are reported in Table 2. The median length of hospital stay was 14 (13–19) days. An overall complication rate of 25.3% was observed. Major surgical complications (defined as anastomotic leak, postoperative abdominal abscess, fascial dehiscence, peritonitis, sepsis, secondary hemorrhage, and relaparotomy for any reason during the postoperative course) were observed in 10.6% of the patients. The number of overall (*p* = 0.126) and major (*p* = 0.238) postoperative complications between total and subtotal gastrectomies was not significantly different. Postoperative 30 day mortality rate and in-hospital mortality rate were 4.7% and 5.7%, respectively. The median follow-up time was 27 (10–70) months with an estimated 5 year survival rate of 53.7%. OS was significantly different across American Joint Committee on Cancer/Union for International Cancer Control (AJCC/UICC) stages (*p* < 0.001). Median OS of all patients was 61 (95% CI: 50.05–71.95) months. Figure 1 shows the corresponding Kaplan–Meier survival curves. Operating times and intraoperative blood loss were not documented before 2004 and 2005, respectively. However, when patients with data on operating time (n = 132) were analyzed, the median operating time was 248 (213–298) min. For patients with data on intraoperative blood loss (n = 82), a median blood loss of 300 (200–600) mL was observed.

To investigate the impact of postoperative complications on OS, patients were stratified into two cohorts: one cohort consisting of all patients in the study and one cohort comprising only patients without complication-related postoperative deaths (30 day mortality and in-hospital mortality). Univariable and multivariable Cox regression analyses were performed to determine the parameters that might influence OS.

### 2.3. Risk Factors for Decreased Overall Survival in the Entire Cohort

The impact of clinically relevant variables on OS of all patients in the study is shown in Table 3. Overall complications (*p* < 0.001), major surgical complications (*p* = 0.003) and anastomotic leak (*p* < 0.001) were significant risk factors for decreased OS in univariable analysis. Other significant risk factors in univariable analysis were higher pT (*p* < 0.001), higher pN (*p* < 0.001), and higher AJCC/UICC stages (*p* < 0.001), older patient age (*p* < 0.001), earlier year of surgery (*p* = 0.003), non-antropyloric compared to antropyloric tumor location (*p* = 0.025), multivisceral resection (*p* = 0.041), splenectomy (*p* = 0.012), additional intestinal resections (*p* < 0.001), and additional pancreatic procedures (*p* = 0.010). When multivariable analysis was performed, the occurrence of overall postoperative complications was an independent risk factor for decreased OS (*p* < 0.001) together with advanced pT (*p* < 0.001) and pN (*p* < 0.001) stages, older patient age (*p* < 0.001), and earlier year of surgery (*p* < 0.001). For patients with data on operating time and intraoperative blood loss were available, neither parameter had a significant impact on OS in univariable analysis (operating time: *p* = 0.327, HR 0.997; blood loss: *p* = 0.147; HR 0.999).

### 2.4. Risk Factors for Decreased Survival after Exclusion of Early Postoperative Mortality

For this subgroup analysis (n = 1042), patients with complication-related early postoperative mortality (30 day mortality and in-hospital mortality) were excluded (Table 4). Overall complications (*p* = 0.198), major surgical complications (*p* = 0.832) and anastomotic leak (*p* = 0.396) did not reach statistical significance as risk factors for decreased OS in univariable analysis. Significant risk factors in univariable analysis were advanced pT (*p* < 0.001), pN (*p* < 0.001), and AJCC/UICC (*p* < 0.001) stages, older patient age (*p* < 0.001), proximal tumor location (*p* = 0.008), multivisceral resection (*p* = 0.007), splenectomy (*p* = 0.004), additional intestinal resections (*p* < 0.001), additional pancreatic procedures (*p* = 0.006), diffuse histologic type according to the Laurén classification (*p* = 0.046), and performance of a total vs. subtotal gastrectomy (*p* = 0.027). Earlier year of surgery did not reach statistical significance on the α = 0.050 level in univariable analysis but showed a trend towards shorter OS (*p* = 0.064) and was therefore included in the multivariable analysis. In a multivariable Cox regression analysis, independent risk factors associated with decreased OS were advanced pT (*p* < 0.001) and pN (*p* < 0.001) stage, older patient age (*p* < 0.001), earlier year of surgery (*p* = 0.010), proximal tumor location (*p* = 0.040), and diffuse histologic type according to the Laurén classification (p = 0.013). For patients with data on operating time and intraoperative blood loss available, neither parameter had a significant impact on OS in univariable analysis (operating time: *p* = 0.821, HR 0.999; blood loss: *p* = 0.290; HR 0.999).

### 2.5. Neoadjuvant and Adjuvant Treatment

Administration of neoadjuvant and adjuvant chemotherapy was not documented in the database before the year 2005 and 2007, respectively. When patients treated with neoadjuvant (n = 106) and adjuvant (n = 75) therapy were investigated, neither treatment had a significant impact on OS in univariable analysis, neither before (neoadjuvant: *p* = 0.104, HR 0.466; adjuvant: *p* = 0.698, HR 1.229) nor after exclusion of in-hospital mortality (neoadjuvant: *p* = 0.214, HR 0.550; adjuvant: *p* = 0.501, HR 1.461).

### 2.6. Subgroup Analysis of AJCC/UICC Stages after Exclusion of Early Postoperative Mortality

As the impact of postoperative complications on OS might vary between patients with different tumor stages, subgroup analyses for AJCC/UICC stages I–IV were performed after exclusion of early postoperative mortality. For patients with AJCC/UICC stage I (n = 489) and IV (n = 57), neither overall complications (*p* = 0.452, HR 0.843; *p* = 0.669, HR 1.219), nor major postoperative complications (*p* = 0.274, HR 0.698; *p* = 0.521, HR 0.519) or anastomotic leak (*p* = 0.420, HR 0.624; *p* = 0.743, HR 0.713) reached statistical significance in univariable analysis. For patients with AJCC/UICC stage II (n = 249) no influence of overall complications (*p* = 0.100), anastomotic leak (*p* = 0.234) nor major surgical complications (*p* = 0.061) on OS was observed in univariable analysis. When multivariable analysis was performed (Appendix Table A1), only the type of gastrectomy (*p* = 0.037) and the year of surgery (*p* = 0.001) remained in the model as factors with significant impact on OS. For patients with AJCC/UICC stage III (n = 246), no significant influence of overall complications (*p* = 0.288), major surgical complications (*p* = 0.705), or anastomotic leak (*p* = 0.097) on OS was observed in univariable analysis. When multivariable analysis was performed (Appendix Table A2), older patient age (*p* = 0.012) was the only significant factor associated with OS.

## 3. Discussion

This study examined factors associated with OS after radical resection for gastric cancer over a time period of more than four decades in a cohort of 1107 consecutive patients at a European university surgical center. In our previous work, we investigated trends in postoperative morbidity, mortality, and failure to rescue in this study population (Christian Galata, Ulrich Ronellenfitsch, Susanne Blank, Christoph Reissfelder, Julia Hardt: Postoperative morbidity and failure to rescue in surgery for gastric cancer: A single center analysis of 1107 patients from 1972 to 2014; submitted and under review, November 2019). Here, we aimed to investigate risk factors for decreased OS with particular consideration for the role of postoperative overall complications and major surgical complications.

The main finding of this study was that postoperative complications had a significant impact on OS in the entire cohort in both univariable and multivariable analysis. However, when complication-related early postoperative deaths (30 day mortality and in-hospital mortality) were excluded, a statistically significant effect of postoperative complications on patient survival was no longer observed. This also holds true for all AJCC/UICC stages subgroups.

The clinicopathological features of the patients in our study are comparable to those of other long-term evaluations [5]. Multivariable analysis identified overall complications, advanced pT stage, advanced pN stage, older patient age, and earlier year of surgery as risk factors for decreased OS in the entire cohort. After exclusion of patients with early postoperative mortality, multivariable analysis rendered advanced pT stage, advanced pN stage, older patient age, earlier year of surgery, proximal tumor location, and diffuse histologic type according to the classification of Laurén as risk factors for decreased OS. The risk factors identified in our cohort are in line with those reported by other authors [6,7]. Possible reasons for the association of an earlier year of surgery with worse OS are improvements in surgical strategy, perioperative management, and oncologic therapy. Notably, when complication-related postoperative deaths were excluded neither major surgical complications nor anastomotic leak or overall complications were significant risk factors for decreased OS in univariable and multivariable analysis.

Recently, a number of studies have investigated the impact of postoperative complications on OS after surgery for gastric cancer, but the way in which the results have been reported is inconsistent. Some studies excluded patients with in-hospital mortality [6,8,9,10,11], while others did not exclude in-hospital mortality, thus potentially increasing the chance of detecting significant correlations between postoperative morbidity and patient survival [7,12,13,14,15,16]. A meta-analysis by Wang et al. found postoperative complications to correlate with poor prognosis after radical gastrectomy [4]. However, this effect was markedly weaker when the authors analyzed the subgroup of studies in which patients with in-hospital mortality were excluded.

In general, studies on this topic usually either include or exclude early postoperative mortality. Data on the effects of including or excluding surgery-related mortality are rarely reported for the same study population. In our analysis, we show that postoperative complications have a significant impact on OS if complication-related early postoperative deaths are not excluded. After exclusion of 30 day and in-hospital mortality, neither overall complications nor major surgical complications or anastomotic leak showed a significant effect on OS in the entire cohort. Likewise, no significant effect of these parameters on OS was observed in multivariable analysis when subgroup analyses of AJCC/UICC stages were performed. Thus, our data suggest that the identification of postoperative complications as a risk factor for inferior oncologic outcomes after radical resection for gastric cancer may have been overestimated depending on whether and how early postoperative mortality is excluded. These results are not in line with the study results by Jin et al. who report that postoperative complications remained an independent risk factor for decreased OS after curative resection for gastric cancer, even after exclusion of patients who died within 30 days postoperatively. Moreover, patients who experienced postoperative complications were 50% less likely to receive adjuvant therapy. The combination of postoperative complications and failure to receive adjuvant therapy increased the risk of death more than twice compared to patients without postoperative morbidity who successfully underwent adjuvant therapy [17]. To summarize, the currently available evidence on the impact of postoperative morbidity on long-term oncologic outcome is still heterogeneous.

Some limitations of this study must be mentioned. The study was retrospective and, thus, the inherent potential for misclassification may limit the validity of our data. In addition, the median OS exceeded the median follow-up which may limit the conclusions. There may be confounding variables that were not available for analysis, in particular preoperative ECOG (Eastern Cooperative Oncology Group) performance status, which might impact patient outcomes. However, since we investigated a cohort that underwent radical resection with curative intent, it may seem justified to assume that the vast majority of our patients were in a general condition that allows for extensive upper abdominal surgery. This is supported by the data of patients for whom ASA grading was available, which were categorized as ASA II and III in 82.7% of the cases. No continuous documentation of the extent of LAD, the number of harvested lymph nodes or the administration of perioperative chemotherapy was available over the long period covered in this study, but data of patients for whom the extent of LAD or the number of harvested lymph nodes were available indicate that D2 LAD with adequate extent was performed for the vast majority of patients in our cohort. When patients with data on neoadjuvant and adjuvant therapy were analyzed, neither of the treatments had a significant impact on OS in univariable analysis, neither before nor after exclusion of in-hospital mortality. These data must be interpreted cautiously, as the small sample size and lack of data on the completeness of chemotherapy and chemotherapy regimens used may have led to a bias. Neoadjuvant treatment, which was introduced at our institution in 2005, was not found to increase postoperative morbidity or mortality in several other studies [18,19], but in itself improves the prognosis of patients with locally advanced gastric cancer [20]. Concerning the extent of LAD, a more aggressive (D 1–3 or D 1–2 instead of D1 alone) surgical procedure might be associated with an increase in postoperative morbidity [21,22]. On the other hand, a more radical lymph node dissection (D 1–2 vs. D1 alone) has been shown to prolong OS [23,24].

## 4. Materials and Methods

### 4.1. Ethics Approval

Ethics board approval was obtained from the Medical Ethics Commission II of the Medical Faculty Mannheim, Heidelberg University, Mannheim, Germany (2019–849R). All patient data used in this analysis were completely anonymized. The study was performed according to the Declaration of Helsinki.

### 4.2. Patients

**Figure 2:**
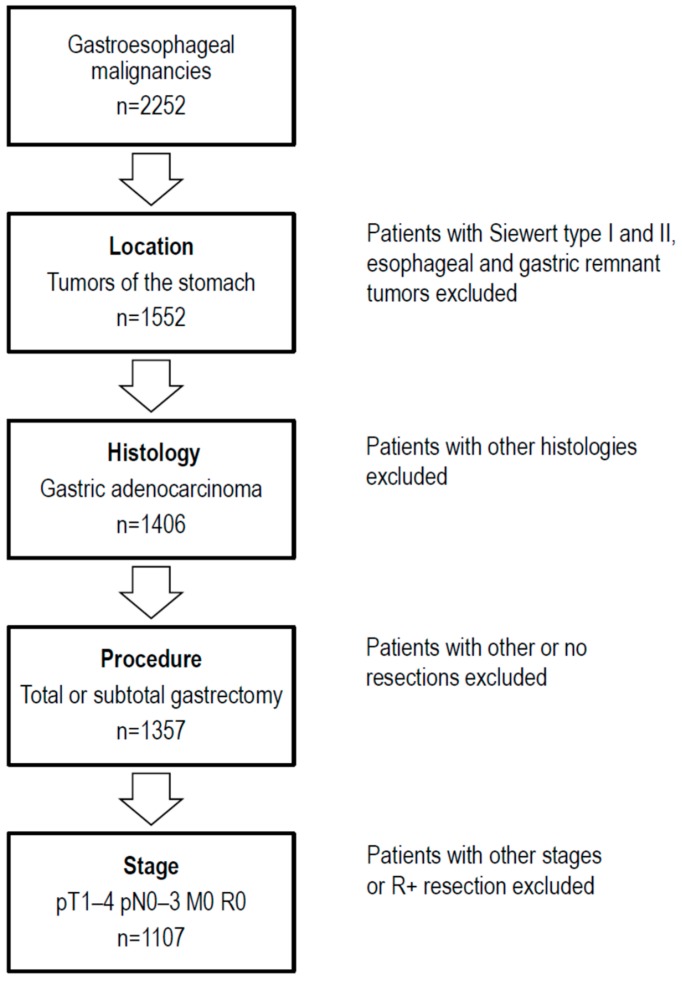
Flow chart of the study population.

A retrospective analysis of our institutional database for surgical patients with gastroesophageal malignancies was performed. Medical records of 2252 consecutive patients operated on between October 1972 and February 2014 were examined, and 1107 patients with M0 gastric cancer who underwent R0 resection were included in the analysis. Patients with Barrett carcinoma, gastric remnant cancer, atypical gastric resections, esophageal resections or pT0 stage on final histology workup were excluded. Tumors of the subcardial stomach (Siewert type III) were included, whereas esophagogastric junctional adenocarcinomas (Siewert type I and II) were excluded, as these are classified and staged according to the esophageal scheme in the current AJCC/UICC system [25]. A flow chart of the study population is shown in Figure 2.

### 4.3. AJCC/UICC Stages

For gastric cancer patients operated on between 1972 and 2001, AJCC/UICC stages according to the 5th edition of the AJCC/UICC staging system were available. The 6th and 7th edition of the AJCC/UICC classification were used from 2002 until 2009 and from 2010 until 2014, respectively. Before analysis, all patients in this study were restaged according to the 6th edition of the AJCC/UICC staging system for gastric cancer.

### 4.4. Postoperative Complications

Data on postoperative complications were extracted from the database, where they had been documented based on medical records. Major surgical complications were defined as one of the following events during the postoperative course: anastomotic leak (including duodenal stump insufficiency), postoperative abdominal abscess, fascial dehiscence, peritonitis, sepsis, secondary hemorrhage, and relaparotomy for any reason. When multiple complications occurred, the most severe complication was used for classifying if a patient had major surgical complications. Complication-related postoperative mortality is presented as early postoperative (30 day) and in-hospital mortality.

### 4.5. Follow-Up and Overall Survival

Follow-up in the database was based on medical records and direct contact with the patient or with the treating physicians. OS time was defined as the interval from surgery to death or latest time point the patient was known to be alive.

### 4.6. Statistical Analysis

Mean and standard deviations were calculated for quantitative variables. Qualitative variables were quoted as absolute numbers and relative frequencies. Median and interquartile range (IQR) are presented for skewed or ordinal scaled parameters. All statistical tests for the comparison of two groups were two-tailed. In general, a test result was considered statistically significant if *p* < 0.050. For qualitative variables, a Fisher’s exact test was used. Univariable and multivariable Cox regression analyses were performed to identify factors that might influence OS. Variables reaching a significance level of α = 0.100 in univariable Cox regression analyses were used as covariates in multivariable Cox regression analyses. In the multiple analyses, the backward stepwise selection based on the probability of the Wald statistic was used, and a significance level of α = 0.050 was chosen to detect several parameters that might influence the outcome. Hazard ratios in the multiple analyses are presented together with their 95% CI. The Kaplan–Meier method was used to present survival data and the log-rank test was used to compare survival distributions. Statistical analyses were performed using the SAS statistical analysis software (release 9.4, Cary, NC, USA).

## 5. Conclusions

In summary, our data support the evidence that postoperative complications are a significant risk factor for poor OS in patients undergoing radical resection for gastric cancer. However, our study shows that this was an effect caused by complication-related early postoperative mortality. Indeed, postoperative complications did not have an impact on OS in patients who were successfully rescued from postoperative overall or major surgical complications.

## Figures and Tables

**Table 1:** Patient characteristics.

Variable	n or Median	% or IQR
**Gender**		
Female	499	45.1
Male	608	54.9
**Age (years)**	65	56–72
**Gastrectomy**		
Total	521	47.1
Subtotal	586	52.9
**Multivisceral resection**	503	45.9
Splenectomy	375	34.2
Cholecystectomy	120	10.9
Intestinal resection	29	2.6
Pancreatic procedure	29	2.6
Hepatic procedure	20	1.8
**Tumor location**		
Non-antropyloric	674	60.9
Antropyloric	433	39.1
**Laurén classification**		
Diffuse	442	40.9
Non-diffuse	638	59.1
**Signet-ring cell carcinoma**	306	27.6
**pT stage**		
T1	267	24.1
T2	579	52.3
T3	211	19.1
T4	50	4.5
**pN stage**		
N0	530	47.9
N1	366	33.1
N2	181	16.4
N3	29	2.6
**AJCC/UICC stage**		
IA	229	20.7
IB	286	25.9
II	263	23.8
IIIA	187	16.9
IIIB	76	6.9
IV	65	5.9

IQR: interquartile range; AJCC: American Joint Committee on Cancer; UICC: Union Internationale Contre le Cancer.

**Table 2:** Patient outcomes.

Variable	n or Median	% or IQR
**Length of stay (days)**	14	13–19
**Overall complications**	277	25.3
**Major surgical complications**	116	10.6
Anastomotic leak	50	43.1
Abdominal abscess/fascial dehiscence	36	31.0
Secondary hemorrhage	18	15.5
Relaparotomy (other than above)	6	5.2
Relaparotomy (not specified)	5	4.3
General sepsis (not specified)	1	0.9
**Postoperative mortality**		
30 day mortality	52	4.7
In-hospital mortality	63	5.7
**Median follow-up (months) ^a^**	27	10–70
**5 year survival rate ^a^**		53.7
**5 year survival rate ^b^**		50.1

**^a^** Patients with in-hospital mortality excluded **^b^** All patients.

**Table 3:** Factors associated with OS (all patients).

Variable	Univariable		Multivariable (*n* = 935)	
	*p* Value	Hazard Ratio	*p* Value	Hazard Ratio (95% CI)
Overall complications	<0.001	1.904	**<0.001**	1.968 (1.617–2.396)
Major surgical complications	0.003	1.506	0.327	
Anastomotic leak	<0.001	2.252	0.360	
pT stage	<0.001	1.892	**<0.001**	1.604 (1.414–1.819)
pN stage	<0.001	1.726	**<0.001**	1.518 (1.356–1.698)
AJCC/UICC stage	<0.001	1.485	0.124	
Age	<0.001	1.021	**<0.001**	1.020 (1.011–1.029)
Year of surgery	0.003	0.984	**<0.001**	0.973 (0.963–0.984)
Tumor location (distal vs. proximal)	0.025	1.236	0.289	
Multivisceral resection	0.041	1.204	0.135	
Splenectomy	0.012	1.263	0.149	
Intestinal resection	<0.001	2.487	0.096	
Pancreatic procedure	0.010	1.896	0.335	
Gender (male vs. female)	0.297	1.099		
Laurén type (diffuse vs. non-diffuse)	0.114	0.864		
Signet-ring cell carcinoma	0.367	0.911		
Gastrectomy (total vs. subtotal)	0.149	0.877		
Hepatic procedure	0.806	1.098		
Cholecystectomy	0.984	1.003		

AJCC: American Joint Committee on Cancer; OS: Overall survival; UICC: Union Internationale Contre le Cancer. *p* values in bold type indicate statistical significance in multivariable analysis.

**Table 4:** Factors associated with OS (in-hospital mortality excluded).

Variable	Univariable		Multivariable (n = 862)	
	*p* Value	Hazard Ratio	*p* Value	Hazard Ratio (95% CI)
pT stage	<0.001	2.022	**<0.001**	1.638 (1.428–1.879)
pN stage	<0.001	1.830	**<0.001**	1.578 (1.399–1.779)
AJCC/UICC stage	<0.001	1.546	0.052	
Age	<0.001	1.019	**<0.001**	1.022 (1.012–1.032)
Year of surgery	0.064	0.990	**0.010**	0.985 (0.973–0.996)
Tumor location (distal vs. proximal)	0.008	1.308	**0.040**	1.236 (1.009–1.515)
Multivisceral resection	0.007	1.297	0.893	
Splenectomy	0.004	1.333	0.679	
Intestinal resection	<0.001	2.645	0.117	
Pancreatic procedure	0.006	2.061	0.557	
Laurén type (diffuse vs. non-diffuse)	0.046	0.821	**0.013**	0.771 (0.629–0.946)
Gastrectomy (total vs. subtotal)	0.027	0.807	0.170	
Overall complications	0.198	1.168		
Major surgical complications	0.832	1.038		
Anastomotic leak	0.396	1.250		
Signet-ring cell carcinoma	0.403	0.911		
Gender (male vs. female)	0.401	1.085		
Hepatic procedure	0.511	1.285		
Cholecystectomy	0.762	1.054		

AJCC: American Joint Committee on Cancer; OS: Overall survival; UICC: Union Internationale Contre le Cancer. *p* values in bold type indicate statistical significance in multivariable analysis.

## Appendix A

**Table A1:** Factors associated with OS in patients with AJCC/UICC stage II (in-hospital mortality excluded).

Variable	Univariable		Multivariable (n = 218)	
	*p* Value	Hazard Ratio	*p* Value	Hazard Ratio (95% CI)
Year of surgery	0.005	0.970	**0.001**	0.963 (0.941–0.986)
Gastrectomy (total vs. subtotal)	0.096	0.726	**0.037**	0.665 (0.453–0.976)
Intestinal resection	0.041	3.358	0.057	
Major surgical complications	0.061	1.739	0.079	
Splenectomy	0.089	1.375	0.356	
Overall complications	0.100	1.447		
Anastomotic leak	0.234	1.724		
pT stage	0.691	0.901		
pN stage	0.691	1.110		
Tumor location (distal vs. proximal)	0.890	0.974		
Laurén type (diffuse vs. non-diffuse)	0.196	0.784		
Signet-ring cell carcinoma	0.644	1.113		
Gender (male vs. female)	0.471	1.145		
Age	0.350	1.008		
Multivisceral resection	0.243	1.248		
Pancreatic procedure	0.739	0.788		
Hepatic procedure	0.974	0.981		
Cholecystectomy	0.962	0.984		

AJCC: American Joint Committee on Cancer; OS: Overall survival; UICC: Union Internationale Contre le Cancer. *p* values in bold type indicate statistical significance in multivariable analysis.

**Table A2:** Factors associated with OS in AJCC/UICC stage III (in-hospital mortality excluded).

Variable	Univariable		Multivariable (n = 196)	
	*p* Value	Hazard Ratio	*p* Value	Hazard Ratio (95% CI)
Age	0.012	1.023	**0.012**	1.023 (1.005–1.041)
Splenectomy	0.086	1.351	0.061	
Anastomotic leak	0.097	2.018	0.069	
Overall complications	0.288	1.246		
Major surgical complications	0.705	1.122		
pT stage	0.102	1.315		
pN stage	0.670	0.938		
Tumor location (distal vs. proximal)	0.220	1.266		
Laurén type (diffuse vs. non-diffuse)	0.275	0.825		
Signet-ring cell carcinoma	0.733	1.067		
Gender (male vs. female)	0.445	0.874		
Year of surgery	0.340	0.990		
Gastrectomy (total vs. subtotal)	0.358	0.850		
Multivisceral resection	0.161	1.282		
Intestinal resection	0.495	1.366		
Pancreatic procedure	0.819	0.908		
Hepatic procedure	0.551	0.549		
Cholecystectomy	0.811	1.082		

AJCC: American Joint Committee on Cancer; OS: Overall survival; UICC: Union Internationale Contre le Cancer. *p* values in bold type indicate statistical significance in multivariable analysis.
